# Designing and evaluating the acceptability of a psychosocial and socioeconomic support package for people with drug-resistant tuberculosis in Johannesburg, South Africa

**DOI:** 10.1371/journal.pone.0343154

**Published:** 2026-03-03

**Authors:** Ndiviwe Mphothulo, Marian Loveday

**Affiliations:** 1 School of Public Health and Nursing, University of KwaZulu Natal, Durban, South Africa; 2 HIV and other Infectious Diseases Research Unit (HIDRU), South African Medical Research Council, CAPRISA-MRC HIV-TB Pathogenesis and Treatment Research Unit, Durban, South Africa; University of the Witwatersrand Johannesburg, SOUTH AFRICA

## Abstract

Drug-resistant tuberculosis (DR-TB) is a global health problem that presents multifaceted challenges to people living with the disease. These challenges lead to sub-optimal adherence in some DR-TB patients who are then not cured of their TB. Besides the challenges associated with taking treatment, many patients with DR-TB also have to contend with psychosocial and socioeconomic challenges. The objective of this study was to develop a psychosocial and socioeconomic intervention for people with DR-TB in Johannesburg, South Africa, and evaluate if they find it acceptable. Guided by the Behaviour Change Wheel (BCW) and Perceptions and Practicalities Approach (PaPA) frameworks, and utilising a participatory research approach, We developed a support package with input from a qualitative needs assessment with DR-TB patients (n = 16) and family members (n = 8) and input from various stakeholders (n = 18), (health managers, clinicians and officials from social security departments). The support package was then evaluated for acceptability by patients who had successfully completed DR-TB treatment (n = 13) and their families (n = 6), using an exploratory qualitative method. Both successfully treated DR-TB patients and their family members found the intervention to be acceptable and believed it will reduce the barriers to retention in care that they faced during their treatment journey.

## 1. Introduction

Tuberculosis (TB) is a major public health problem that poses a significant threat to global health. In 2023, the global TB incidence rate was reported at 133 per 100,000 population, while the African region had an estimated incidence rate of 208 per 100,000 [1]. This represents a significant increase from the 7.5 million newly diagnosed TB cases reported globally in 2022, and 7.1 million in 2019, with 8.2 million newly diagnosed cases reported globally in 2023 [1]. The World Health Organization (WHO) estimated South African (SA) incidence rate of 468 per 100,000, surpasses global and African regional rates [1]. The South African National Prevalence Survey conducted between 2017 and 2019 reported a prevalence of 852 TB cases per 100,000 people [2]. Drug resistant Tuberculosis (DR-TB) occurs when *Mycobacterium tuberculosis* bacteria become resistant to the drugs used to treat TB [3]. In SA an estimated 13 000 people developed DR-TB in 2023 [1]. In 2021, the last year of available report, global treatment success rates for people with DR-TB were 68% and 62% for SA [1] falling short of the 75% success rate target set by the WHO [4]. In addition to having to cope with the disease, treatment regimens with numerous and often severe adverse effects, many DR-TB patients have to contend with psychosocial and socioeconomic challenges, which compromise their ability to remain in care for the full duration of treatment.

Despite advances in the microbiological and clinical aspects of DR-TB management, there has been limited research on the contribution of psychosocial and socioeconomic factors to unsuccessful DR-TB treatment outcomes and how these could be addressed. In this study, we developed a psychosocial and socioeconomic support package to facilitate retention in care of people with DR-TB, which was then evaluated for acceptability by cured former DR-TB patients who had initially struggled to adhere to treatment and their families.

## 2. Materials and methods

### Study design

The study was conducted over 3 phases in Johannesburg, Gauteng Province (GP), South Africa. Phase 1 was a qualitative needs analysis with DR-TB patients who initially struggled to adhere to treatment and their families to determine barriers and facilitators to retention in care and recommendations on suggestions to improve retention in care. Phase 2 was the design of the support package by various stakeholders, and Phase 3 was an evaluation of acceptability of the intervention by the same DR-TB patients, who were subsequently cured, and their families involved in Phase 1.

### Qualitative needs analysis (Phase 1)

The needs analysis was guided by the qualitative phenomenology approach [5]. Using Semi Structured Interviews (SSIs) people with DR-TB (n = 16) their family members (n = 8) were interviewed. There was one Focus Group Discussion (FGD) with 10 participants, 6 DR-TB patients and 4 family members. DR-TB patients who had disengaged from DR-TB care for ≥ 45 days and their family members were selected from five DR-TB facilities which were purposefully selected due to their high loss to follow-up (LTFU) rate and represented both large and small facilities.

### Design of the support package (Phase 2)

Through Collaborative Change Research, Evaluation, and Design (CCRED) [6, 7] the collaborative process involved a series of in-person and virtual meetings with a diverse group of stakeholders, known as the Intervention Development Group (IDG). The IDG comprised district, subdistrict, and health facility managers, nurses and doctors directly involved in the management of DR-TB patients, Ward Based Outreach Teams (WBOT) managers, and officials from social security government departments; the Department of Social Department (DSD) and the South African Social Security Agency (SASSA) [8]. The WBOTs are community health workers who are attached to a primary healthcare facility, and operate within a defined area providing promotive and preventive health services. The diverse composition of the IDG was to ensure comprehensive insights and multiple perspectives on the intervention package [9].

**Conceptual and theoretical frameworks** The design of the intervention was informed by two theoretical frameworks: Behaviour Change Wheel (BCW) [10], and the Perceptions and Practicalities Approach (PaPA) [11]. The BCW provided a structured approach to understanding behaviour change, while PaPA guided our consideration of patients’ perceptions and practical realities. The motivation for the use of these frameworks was that the support package will enhance patient experience, facilitate retention in care and improve treatment outcomes as illustrated by the conceptual framework (See S1 File).

### Evaluation of acceptability of the intervention (Phase 3)

For this study component, we employed a longitudinal sampling approach, re-recruiting participants from Phase 1. Of the 16 DR-TB patients in Phase 1, 14 (87.5%) achieved successful treatment outcomes (cured), one passed away in a motor vehicle accident. Consequently,13 former DR-TB patients (81.3%) were available for interviews. In Phase 1, eight family members participated in interviews. in Phase 3, we interviewed six family members.

### Study setting

The study was conducted at Johannesburg, Gauteng Province (GP) in South Africa, from 03 May 2023–06 January 2025. DR-TB treatment in Johannesburg is delivered at 9 facilities. One of these is a specialised DR-TB treatment hospital which provides both inpatient and outpatient care for all types of DR-TB including rifampicin/multidrug resistant TB (RR/MDR-TB), pre-extensively drug resistant TB (pre-XDR-TB), and extensively drug resistant TB (XDR-TB). RR/MDR-TB is defined as *Mycobacterium tuberculosis* with resistance to rifampicin. It includes MDR-TB (resistance to both isoniazid and rifampicin) and rifampicin mono-resistant TB (susceptibility to isoniazid). Pre-XDR TB is MDR-TB with resistance to any fluoroquinolone, and XDR-TB is TB resistant to rifampicin, plus any fluoroquinolone and one further group A drug, bedaqualine or linezolid [12]. The eight other health facilities offer outpatient RR/MDR-TB treatment only. Initial DR-TB diagnosis is made in local health facilities across Johannesburg, and on diagnosis, patients are referred to the DR-TB treatment facility closest to their home for initiation of treatment and ongoing management. DR-TB treatment is free, and free transport is provided from local clinics to DR-TB treatment facilities. Each month, patients are expected to return to their DR-TB treatment facility to monitor their response to treatment and collect medication for the following month. Patients with successful DR-TB treatment outcomes are discharged from treatment, but are asked to return 6-monthly to assess whether any signs and symptoms of TB have re-emerged and sputum microscopy and culture are performed.

### Characteristics of study participants

Table 1 describes the characteristics of study participants (Table 2).

**Table 1:** Table of characteristics of study participants involved in each phase of study.

Study Phase	Participant Category	Number of Participants	Characteristics of participants
1	DR-TB Patients	16	• 13 on short regimen (9 months) • 3 on long regimen (18 months)
1	Family members	8	• 5 females • 3 males
2	Stakeholders	18	• 7 WBOTs managers • 3 Medical doctors • 2 DR-TB nurses • 1 each: DR-TB facility manager, JDH HAST directorate representative, sub district manager, NTP representative, DSD representative, and SASSA representative.
3	Cured DR-TB patients from phase 1 of the study	13	• 6 females • 7 males
3	Family members from phase 1 of the study	6	• 4 females • 2 males

DR-TB (Drug-Resistant tuberculosis JDH (Johannesburg District Health) NTP (National Tuberculosis Program) DSD (Department of Social Development) SASSA (South African Social Security Agency), WBOTs (Ward Based Outreach Teams).

**Table 2:** Table of characteristics of the cured former DR-TB patients in phase 3.

Study ID	Gender	*Resistance Pattern	Duration to complete DR-TB treatment
#P1	Female	RR-TB	12 months
#P2	Female	MDR-TB	13 months
#P3	Female	MDR-TB	18 months
#P4	Male	MDR-TB	11 months
#P5	Female	Pre-XDR-TB	20 months
#P6	Male	MDR-TB	11 months
#P7	Male	MDR-TB	11 months
#P8	Female	MDR-TB	13 months
#P9	Male	XDR-TB	21 months
#P10	Male	MDR-TB	11 months
#P11	Female	RR-TB	12 months
#P12	Male	MDR-TB	12 months
#P13	Male	RR-TB	13 months

*Resistance Pattern: Type of DR-TB.

### Family members are designated #P14-#P19

RR-TB (Rifampicin Resistant TB), MDR-TB (Multiple Drug Resistant TB), Pre-XDR (Pre-Extreme Drug Resistant TB), XDR-TB (Extreme Drug Resistant TB)

### Ethical considerations

Ethical approvals for all study phases were granted by the Biomedical Research Ethics Committee (BREC), BREC/00004973/2022, of the University of KwaZulu Natal, and permission to conduct the study was also obtained from the Gauteng Department of Health. Ethics approval for Phases 1 and 2 of the study was approved on 27 March 2023 and expired on 26 March 2024. A new ethics approval was obtained for phase 3, which commenced 21 November 2024 (renewal approval starting from 12 November 2024). Written informed consent to participate in the study was received from the participants. Participants received detailed information about the study, and their questions were addressed before signing the consent form, confirming their voluntary participation. The consent included participation in the SSI or FGD and digital audio recording, the voluntary terms of involvement in the study and the assurance of confidentiality and anonymity. Participant anonymity was maintained by identifying each patient using a unique identification number. This study was conducted in accordance with the declaration of Helsinki (2013) and the principles of good clinical practice.

### Data collection

#### Needs analysis.

This study component has been previously described [5].

### Support package development

Four FGDs were held: Group 1: WBOT managers, Group 2: DR-TB nurses and health managers, Group 3: DR-TB treating doctors and an official from the National TB Program (NTP), and Group 4: DSD and SASSA officials. All stakeholders were government employees responsible for service delivery, employed by the department of health or SASSA/DSD. The meetings were held between 18 September 2023 and 12 October 2023. The stakeholders were categorised into the four groups based on their expertise and professional affiliations (Appendix 1). One meeting was held in person (Group 1) and the others virtually. Discussion points were identified for each group to facilitate focused discussions, with different groups meeting separately to deliberate on aspects of the intervention they had experience with, i.e., therapy related, health system, socioeconomic related and psychological related factors. To start the meetings, the researcher (NM), gave an overview of the research project, the findings of the literature review and the results of needs analysis. The second part of the meeting were workshops, designed to review the outcomes of the needs analysis and recommendations by DR-TB patients and their families.

Stakeholders made inputs on how the recommendations from the needs analysis could be realised through available resources or innovations and through a process of consensus-building, agreed on recommendations for the support package. At the end of the meeting, the researcher summarised the main recommendations from the group to ensure these were correctly captured and a true reflection of the discussion.

### Support package evaluation

To evaluate the acceptability of the support package, SSIs were used to collect data from cured former DR-TB patients and their family members. Following the piloting of the SSIs, interviews of participants were conducted at venues, or in a private space chosen by participants to ensure confidentiality. The interviews were conducted by a research assistant (TM) who had conducted interviews in phase 1 and had established a relationship with each participant. The interviews were conducted in the language preferred by the patient. The participants were handed the designed intervention package, and each element of the intervention package was explained. Questions in the SSI guided participants to evaluate the designed intervention package, describe which elements of the intervention were perceived to be very useful and whether these interventions would address the challenges to retention in care they experienced during their treatment journey. In addition they were asked if they would like to add refinements to the intervention. The SSIs lasted between 30 and 45 minutes, and were transcribed and translated into English by the researcher (NM).

### Data analysis

#### Needs assessment.

Data analysis for the needs analysis phase has been previously reported [5].

#### Intervention development.

We assessed the findings from the IDG meetings using BCW, [10] framework to identify potential capabilities, opportunities, and motivators (COM-B) that support patient’s adherence to treatment. Additionally we used the PaPA framework, [11] to identify factors that reinforce patients’ and families’ necessity beliefs about the importance of adherence to treatment, dealing with concerns and fear about the treatment, and empowering patients with understanding practicalities of the treatment journey. This analysis helped us to translate recommendations of IDG to interventions that will reduce barriers to retention in DR-TB care.

#### Evaluating acceptability.

Data transcribed from SSIs was read and reread by the researcher, and categorised into themes and subcategories. Data analysis was guided by the WHO recommendations for social support of people with DR-TB, [13] which asserts that availing informational, companionship, emotional and material support offers patient centred support to people with DR-TB. Quotes illustrating each specific theme and subcategory were identified. There was coding and thematic analysis of themes that emerged from the suggestions on refinement of the intervention by participants. These themes were added into Microsoft Excel and organised the four types of social support to DR-TB patient, and a category of “others”. There intervention was refined based on participants’ feedback, with every suggestion provided being accepted and integrated into the final intervention.

## Results

### Themes emerging from needs assessment

The needs assessment phase identified barriers and facilitators to retention in care and recommendations for improving retention and have been published [5]. The key factors which emerged during the SSIs and FGDs for consideration in the package of care were therapy-related, health system, socioeconomic, and psychological factors (S1 Appendix) [5].

### Support package development

The development of the intervention package was informed by the multi-step process involving various stakeholders. The process was consensus driven, where stakeholders deliberated on therapy-related, health system, socioeconomic, and psychological factors, ensuring that the recommendations were feasible within existing policies and resources. Recommendations from the IDG in phase 2 were matched with suggestions from DR-TB patients and family members in phase 1. Insights gathered form FGDs with the diverse stakeholders enriched the support package, resulting in 17 elements (Appendix 2). These elements were categorised based on the target population (DR-TB patients or family members), personnel responsible for implementation, and implementation strategy.

### Evaluation of the support package by former DR-TB patients and their family members

After the deliberations of the IDG the support package was presented to cured former DR-TB patients and their families for evaluation of acceptability (Appendix 3). They reported that the designed support package has addressed almost all their concerns identified in Phase 1 (needs analysis), however they added other elements for refinement of the package. The quotes below illustrate the support of former DR-TB patients and their families for a number of elements in the support package:

### Material support

#### Optimisation of patient transport.

“*The transport system was a major challenge for me, so optimizing it by improving booking and timekeeping by drivers will make a big difference for others.” (#P3, former DR-TB patient)*

*“An improved booking system will eliminate the need for a care giver to go to the clinic a day before the appointment, as it can be confirmed with them telephonically.” (#P15, family member)*

#### Nutritional support.

*“When there is no food at home, I would be weak and skip taking medication. Maybe if had constant supply of food, I would have not taken long to complete my treatment” (#P8, Former DR-TB patient)*

### Informational support

#### Education and counselling.

*“I didn’t know what to expect when I started treatment, so education and counselling would have been really helpful.” (#P5, former DR-TB patient)*

*“Education and counselling about DR-TB would be very helpful as it will empower families with knowledge on what is DR-TB and how to care for a person with DR-TB.” (#P17, family member)*

#### Linking patients with DSD/SASSA.

*“It seems most of us were not aware of availability of food parcels from DSD and the temporary DG from SASSA, some became aware very late. Having someone to help with application for these services will be very helpful” (#P13, former DR-TB patient)*

*“I heard someone advising me about applying for food parcels from DSD, however, I went once to the offices and I never got assistance. This service would be helpful to us as families.” (#P16, family member)*

### Companion support

#### Home visits and adherence support.

*“Having someone to visit me at home and check on my adherence and who understands my challenges would have made me feel more supported.” (#P9, former DR-TB patient)*

*“Home visits would have been very important to us caregivers of people with DR-TB because one got overwhelmed by caring for a person with DR-TB, support and advice will help families to deal with the difficulties of caring for a person with DR-TB.” (#P14, family member)*

### Emotional support

#### DR-TB champions.

*“Getting to understand the experience of treatment journey and information sharing by former patients can be of great value to new patients” (#P2, former DR-TB patient)*

#### Counsellors conducting health promotions.

*“Talks on health education given by counsellors can bring more confidence on how patients can best be empowered to better deal with the disease, and patients will also benefit from teachings on how to cope with the disease” (#P1, former DR-TB patient)*

In addition to the elements listed by the IDG in the support package cured former DR-TB patients and their families added an additional 6 elements: 1) treating newly diagnosed DR-TB patients with respect and dignity; 2) clear communication of microbiology results to newly diagnosed DR-TB patients at local clinic, 3) communication to family members about treatment progress; 4) follow-up of former DR-TB patients at local clinics at the end of treatment for residual side effects; 5) education about TB and DR-TB to general public; and 6) education about TB and DR-TB at schools. Table 3 outlines the final elements of the support package after suggestions of participants in phase 3 of the study.

**Table 3:** The final elements of the support package categorised under social support types.

Social support type	elements of the support package
Information support	1. *Clear communication of microbiology results at diagnosis. 2. Educating and counselling patients and family members about DR-TB and its treatment at diagnosis. 3. Communication on Treatment Progress to DR-TB patients. 4. *Communication on Treatment Progress to family members. 5. Education and poster messages about DR-TB.
Emotional support	1. Appoint DR-TB champions. 2. DR-TB facility Counsellors conducting health promotions. 3. Help patients to cope with side effects. 4. Communicating with DR-TB patients’ place of employment.
Companionship support	1. *Treating newly diagnosed DR-TB patients with respect. 2. Establishing Support groups. 3. Home visits and adherence support.
Material support	1. Optimised transport services. 2. Nutritional support by DR-TB facilities. 3. Linking patients with DSD (nutritional support). 4. Linking patients with SASSA (for financial support).
^#^Others	1. Training SASSA contracted medical doctors about DR-TB. 2. Training WBOTs. 3. Eliminating stigma and discrimination from health facilities. 4. *Education about TB and DR-TB to the general community. 5. *Education about TB and DR-TB to schools. 6. *Follow up at local clinic post treatment. 7. Supporting DR-TB facilities to manage side effects.

*Elements of intervention included after exploratory study in phase 3

# *others: elements targeting other stakeholders, or patients post treatment*

DR-TB (Drug-Resistant tuberculosis JDH (Johannesburg District Health), DSD (Department of Social Development) SASSA (South African Social Security Agency), WBOTs (Ward Based Outreach Teams).

## Discussion

In a three-phase process, a package was developed to provide psychosocial and socioeconomic support to people with DR-TB and their families. Involvement of both DR-TB patients who had initially struggled to remain in care and their families was to ensure that the package is relevant and the involvement of stakeholders from the departments of health and social services optimised its chances of their support and future implementation. [9,14,15]. PR, [15] was utilised through use of PAR [15], involving DR-TB patients and their families in both identifying their needs and evaluating the preliminary package, ensured the package is relevant and addresses their needs; and CCRED [6,7], with various stakeholders in intervention design. The design of the intervention was guided by the use of BCW, [10] and PaPA, [11] frameworks. BCW framework contributed with outlining drivers of patient behaviour in adhering to DR-TB treatment, incorporating strategies to enhance capacity to adhere to treatment, presenting opportunity to facilitate adherence and motivation to complete treatment [10]. Through PaPA framework the intervention integrated patients’ and family’s necessity beliefs about the importance of adhering to treatment, concerns and fears about the treatment, and practicalities of the treatment journey [11].

Psychosocial and socioeconomic support packages have been shown to enhance access to TB prevention, care, and treatment outcomes of patients [16–19]. Studies that have specifically focused on the impact of psychosocial and socioeconomic support to people with DR-TB, have also shown improved DR-TB treatment outcomes [20]. For example, studies in China and India, with socioeconomic conditions similar to those of this study, provided psychosocial and socioeconomic support to DR-TB patients. These interventions included cash handouts, nutritional support, home visits, and treatment support.

In China, Li et al’s provision of cash handouts, nutritional support and providing treatment supporters resulted in tenfold increase in retention in care [21], and in India Teneja et al’s provision of nutritional support, home visits, and information to DR-TB patients showed treatment success rates were significantly higher in the intervention arm (p < 0.03) [22].

Alegria-Flores et al designed and evaluated treatment adherence intervention for DR-TB patients in Peru [23]. Their work highlighted that providing adherence information and motivation, forms of psychosocial support, had positive effects on patients’ adherence. Furthermore, Khanal et al. developed a patient-centred psychosocial support intervention, in Nepal [24], involving DR-TB patients, family members, NTP officials and health workers, similar to this study. Unlike this study, theirs lacked the socioeconomic component and did not assess intervention acceptability. The study’s findings are also consistent with Rai et al’s research in Nepal [25], which developed a psychosocial and socioeconomic intervention for both people with DS-TB and DR-TB. Similar to this study, they engaged people affected by TB in understanding barriers and facilitators to care. However, their stakeholder group differed slightly, comprising NGOs and community leaders instead of family members. Both studies had stakeholders involved in the development phase of the intervention and identified broadly similar psychosocial and socioeconomic components. While Rai et al, [25] further suggested public-private partnerships to enhance TB care, this study emphasised stigma elimination in healthcare facilities and public education. Notably their study did not assess the acceptability of the intervention by TB patients. By also evaluating the acceptability of the intervention package, this study aimed to provide valuable insights into the relevance and potential effectiveness of the designed intervention package. This study, Khanal et al [24], and Rai et al [25], utilised Participatory Research [7, 8, 15,26,27] engaging those who belong to or represent the interests of the people who are the focus of the research in designing the interventions [7].

By involving people with DR-TB and family members in the design and evaluation of acceptability of the intervention package, we aimed to ensure that the intended end users play a prominent role in the design of this support package [28] Through collaborative co-design with stakeholders from the DoH, social security departments, we leveraged their insider knowledge of existing policies and resources necessary to develop feasible solutions [28].

While this study demonstrates acceptability of this psychosocial and socioeconomic intervention, its impact will ultimately depend on successful translation to policy and practice.

Translating research findings into policy can be a complex and challenging process, bridging the gap between research and policy requires more than just evidence-based interventions; it demands effective communication, stakeholder engagement, and policy entrepreneurism [26–29], as this will ensure the buy-in of those who will be involved in its creation, adoption and implementation [9,14,15]. The study’s comprehensive approach combining WHO recommendations for social support for people with DR-TB [4,13], PR [7,14,15,28,30], and assessing intervention acceptabilitym [31], enhances its potential to effectively reduce barriers to adherence reported by DR-TB patients and families in Johannesburg, South Africa [5].

## Conclusion

By prioritising patient-centred care approaches and addressing complex needs of people with DR-TB and their families, this intervention package offers promising strategy for enhancing treatment adherence, retention in care and over-all quality of life of people with DR-TB. Before presentation to the NTP, a pilot study to evaluate the feasibility and cost analysis of the package with possible refinement is necessary. Subsequently, a randomized control trial should be conducted to evaluate the package’s effectiveness.

### Limitations

As a qualitative study, these findings are context-specific and may not be transferable to other settings. Secondly, the study participants were mostly from disadvantaged socioeconomic backgrounds, missing barriers experienced by DR-TB patients from different socioeconomic backgrounds, or those of diverse DR-TB patients. Thirdly, the focus questions included in the SSI and FDG may have introduced some directionality to the discussions, potentially influencing the themes that emerged and the participants’ responses. Fourthly, there could be stakeholder bias, where the involvement of officials from the NTP, SASSA, and DSD in the development process may have introduced bias towards existing systems and policies, and not have thought of new innovations. Furthermore, this paper did not discuss potential implementation challenges, like training and resources constraints, that may impact the implementation and effectiveness of the support package.

## Supporting information

S1 Appendix Stakeholders and Key factors for consideration. (DOCX)

S2 Appendix Support package suggested by IDG. (DOCX)

S3 Appendix Support package presented to participants. (DOCX)

S1 File Conceptual framework of the approach, methods and intended impact of the support package. (PDF)

S2 File Theore-cal framework of acceptability of an interven-on: Sekhon, M., Cartwright, M. & Francis, J.J. (2017). (PDF)

S3 File BCW & PAPA, and Linkage to care. (PDF)

S4 File Figure of BCW Framework and link to IDG recommendations. (PDF)

S5 File Figure of PaPA Framework and link to IDG Recommendations. (PDF)
