# Supplementary material for: Designing and evaluating the acceptability of a psychosocial and socioeconomic support package for people with drug-resistant tuberculosis in Johannesburg, South Africa
Source: PLoS One. 2026 Mar 3;21(3):e0343154. doi: 10.1371/journal.pone.0343154 (PMC12956097; doi:10.1371/journal.pone.0343154)
Supplement: S1 Appendix — (DOCX) [file pone.0343154.s001.docx]

**Appendix 1A: Key factors for consideration** **by stakeholder groups in Intervention Development (Phase 2)**

| **Stakeholder groups** |  |  |  |  |
| --- | --- | --- | --- | --- |
| **Group 1:**  District manager (n=1)  Subdistrict manager (n=1)  DR-TB facility manager (n=1)  DR-TB nurses (n=2) | **Health System related factors:** | **Socioeconomic related factors** | **Psychological related factors** | **Therapy related factors** |
|  | - Counselling and education of DR-TB patients and families at diagnosis of DR-TB. - Improved patient transport booking system. - Improved communication by HCW at DR-TB facilities about treatment progress. - Educating HCW in non DR-TB facilities on ending discrimination against DR-TB patients. - Education and awareness of DR-TB to the general population. | - Assisting DR-TB patients with food parcels for the duration of DR-TB treatment. - Providing more porridge packs supplies by DR-TB facilities. - Assistance in applying for DGs. | - Provision of support through the creation of support groups for DR-TB patients and their families. - Home visits for treatment support and emotional support. - Have a designated person who will partner with the patient throughout the DR-TB treatment journey and support them in addressing their challenges. - Guiding families on how to care for a DR-TB patient. |  |
| **Group 2:**  WBOT managers (n=7) | - Counselling and education, about DR-TB, to families and DR-TB patients on diagnosis of DR-TB. - Improvements to the patient transport booking system. | - Assisting DR-TB patients with food parcels during DR-TB treatment. - Assistance in applying for and receiving temporary DG from SASSA. - Assistance with accessing the UIF for employed DR-TB patients. | - Provision of psychological support through the creation of support groups for DR-TB patients and their families. - Home visits for treatment support and emotional support. - Have a designated person who will partner with the patient throughout the DR-TB treatment journey and support them in addressing challenges. - Guiding families of DR-TB patients on how to care for a patient with DR-TB. |  |
| **Group 3:acility name**  NTP official (n=1)  DR-TB doctors (n=3) | - DR-TB counselling and education to families and DR-TB patients on diagnosis of DR-TB. - Improvements to the patient transport booking system. - Improved communication by HCW at facilities to DR-TB patients about treatment progress. - Educating HCW in other facilities to not discriminate or stigmatize DR-TB patients. - Education and awareness about DR-TB to the general population. | - Assisting DR-TB patients with food parcels for the duration of DR-TB treatment. - Assistance in applying for and receiving temporary DG from SASSA. - Assistance in accessing UIF for employed DR-TB patients. | - Have a designated person who will partner with the patient throughout the DR-TB treatment journey and support them in addressing challenges. - Guiding families of DR-TB patients on how to care for a patient with DR-TB. | - Provision of drugs with lesser side effects. - Reducing pill burden. - Management and treatment of side effects. |
| **Group 4:**  DSD official (n=1)  SASSA official (n=1) |  | - Assisting DR-TB patients with food parcels for the duration of DR-TB treatment. - Assistance in applying for and receiving temporary DG from SASSA. |  |  |

*DR-TB* (*Drug resistant tuberculosis) DSD (Department of Social Development) SASSA (South African Social Security Agency), WBOTs (Ward Based Outreach Teams), DG (Disability Grant), UIF (Unemployment Insurance Fund), HCW (Health Care Workers*
