# Supplementary material for: Designing and evaluating the acceptability of a psychosocial and socioeconomic support package for people with drug-resistant tuberculosis in Johannesburg, South Africa
Source: PLoS One. 2026 Mar 3;21(3):e0343154. doi: 10.1371/journal.pone.0343154 (PMC12956097; doi:10.1371/journal.pone.0343154)
Supplement: S2 Appendix — (DOCX) [file pone.0343154.s002.docx]

**Appendix 2: Support package suggested by stakeholders**

| WBOT Managers | DR-TB Nurses and Health managers | Medical Doctors and the NTP official | SASSA/DSD representatives |
| --- | --- | --- | --- |
| Linking DR-TB patients with WBOT supervisor. | Training of WBOTS to conduct counselling sessions. | Medical doctors to explain treatment plans and progress to DR-TB patients. | Training of WBOTs to help DR-TB patients to apply for food parcels. |
| Appointing a dedicated WBOT team to the DR-TB patient. | DR-TB facility based counsellors to conduct counselling and treatment literacy education. | Development of roadmap documents and posters. | DR-TB patients to receive letters to apply for DG. |
| Counselling and education sessions about DR-TB to patients and family members at their homes. | Setting up of local support groups | DR-TB facility based counsellors to conduct counselling and treatment literacy education. | Training of SASSA contracted doctors about DR-TB, Treatment and side effects. |
| Empowering DR-TB patients and family members to know about DR-TB and its treatment. | WBOTs to book patient transport. Help DR-TB patients and family members to communicate with local clinic, and have contact details to confirm bookings. | Collaboration between DR-TB facilities & tertiary hospitals on treatment of side effects. |  |
| Home visits | Provision of packages of porridge to DR-TB patients irrespective of patient’s BMI. | Communication about the side effects between clinicians and patients. |  |
| Assist with patient transport booking | Provision of month long packages of porridge to DR-TB patients. | Visual aids about side effects. |  |
| WBOT linking DR-TB patients with SASSA and/or DSD | Nurses to conduct treatment literacy education to DR-TB patients at every visit. | DR-TB champions to teach patients about with side effects. |  |
| Visit to patient’s homes or text/phone call reminders to take treatment. | Medical doctors to continuously provide treatment information, treatment plans and engage DR-TB patients as active participants. | A coordinated District plan and training to manage side effects and regular pharmacovigilance meetings in DR-TB facilities. |  |
|  | Development of guidelines on managing visiting DR-TB patients in health facilities | DR-TB facilities to communicate with local clinics about appointments for patients. |  |
|  | Provincial and District campaigns to address stigmatisation of DR-TB patients.. | Referral letters to SASSA, and medical reports for DG applications. |  |

*DR-TB (Drug-Resistant tuberculosis JDH (Johannesburg District Health), DSD (Department of Social Development) SASSA (South African Social Security Agency), WBOTs (Ward Based Outreach Teams).*
