# Supplementary material for: Designing and evaluating the acceptability of a psychosocial and socioeconomic support package for people with drug-resistant tuberculosis in Johannesburg, South Africa
Source: PLoS One. 2026 Mar 3;21(3):e0343154. doi: 10.1371/journal.pone.0343154 (PMC12956097; doi:10.1371/journal.pone.0343154)
Supplement: S3 Appendix — (DOCX) [file pone.0343154.s003.docx]

**Appendix 3: The support package presented to former DR-TB patients and their families for evaluation in Phase 3**

| **Intervention** | **People responsible to implement the intervention** | **People targeted by the intervention** |
| --- | --- | --- |
| 1. Educating and counselling patients and families about DR-TB and its treatment on diagnosis. | - WBOTs, or - Nurses. | - DR-TB patients. - family members. |
| 1. Optimisation of patient transport booking system. | - Local clinic nurses. - WBOTs. - DR-TB facility clinicians. | - DR-TB patients - Family members. |
| 1. Establishing Support groups. | - JDH managers. - WBOTs. - DR-TB facility nurses. | - DR-TB patients. - Family members. |
| 1. Appointment of DR-TB champions. | - JDH managers - DR-TB facility nurses. | - DR-TB patients. |
| 1. DR-TB facility counsellors to conduct DR-TB health promotion. | - DR-TB facility nurses. - DR-TB facility counsellors. | - DR-TB patients. |
| 1. Communication on treatment progress to DR-TB patients. | - DR-TB facility doctors. - DR-TB facility nurses. | - DR-TB patients. |
| 1. Helping patients to cope with side effects. | - DR-TB facility doctors. - DR-TB facility nurses. - WBOTs. | - DR-TB patients. - Family members. |
| 1. Education and Poster messages about DR-TB treatment roadmap and side effects. | - JDH management. - Doctors at DR-TB facilities. | - DR-TB patients. - Family members. |
| 1. Supporting DR-TB facilities to manage side effects. | - JDH management. - Doctors at tertiary hospitals. | - DR-TB patients. |
| 1. Improving nutritional support by DR-TB facilities. | - JDH management. - Dieticians at DR-TB facilities. - DR-TB facility nurses. | - DR-TB patients. |
| 1. Home visits and adherence support. | - WBOTs. | - DR-TB patients. - Family members. |
| 1. Linking DR-TB patients with DSD to address food security challenges. | - JDH. - WBOTs. - DR-TB facility nurses. - Local clinic nurses. | - DR-TB patients. |
| 1. Linking DR-TB patients with SASSA to apply for a temporary DG. | - JDH. - WBOTs. - DR-TB facility nurses. - Local clinic nurses. | - DR-TB patients. |
| 1. Communication with DR-TB patients’ place of employment. | - Medical doctors. - WBOTs. | - DR-TB patients. |
| 1. Training SASSA contracted medical doctors about DR-TB. | - JDH management. | - SASSA contracted medical doctors. |
| 1. Training WBOTs about DR-TB and services offered by DSD and SASSA. | - JDH management. | - WBOTs. |
| 1. Eliminating stigma and discrimination from health facilities. | - JDH. - NTP. | - HCWs. |

*DR-TB (Drug-Resistant tuberculosis JDH (Johannesburg District Health) NTP (National Tuberculosis Program) DSD (Department of Social Development) SASSA (South African Social Security Agency), WBOTs (Ward Based Outreach Teams), DBE (Department of Basic Education)*
