# Supplementary material for: Designing and evaluating the acceptability of a psychosocial and socioeconomic support package for people with drug-resistant tuberculosis in Johannesburg, South Africa
Source: PLoS One. 2026 Mar 3;21(3):e0343154. doi: 10.1371/journal.pone.0343154 (PMC12956097; doi:10.1371/journal.pone.0343154)
Supplement: S1 File — (PDF) [file pone.0343154.s004.pdf]

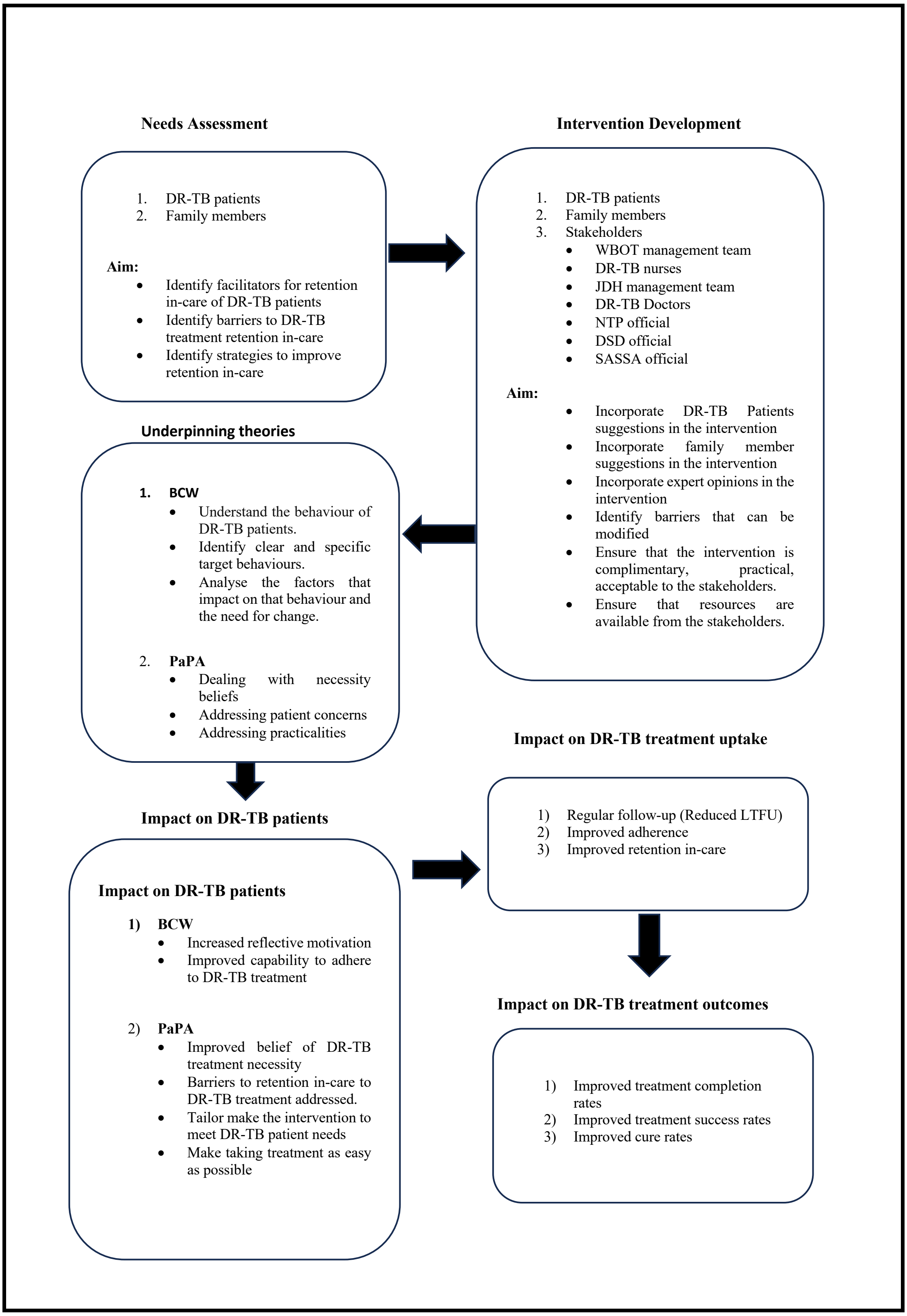

Figure 1: Conceptual framework of the approach, methods and intended impact of the support package
