# Supplementary figures and images for: Designing and evaluating the acceptability of a psychosocial and socioeconomic support package for people with drug-resistant tuberculosis in Johannesburg, South Africa
Source: PLoS One. 2026 Mar 3;21(3):e0343154. doi: 10.1371/journal.pone.0343154 (PMC12956097; doi:10.1371/journal.pone.0343154)

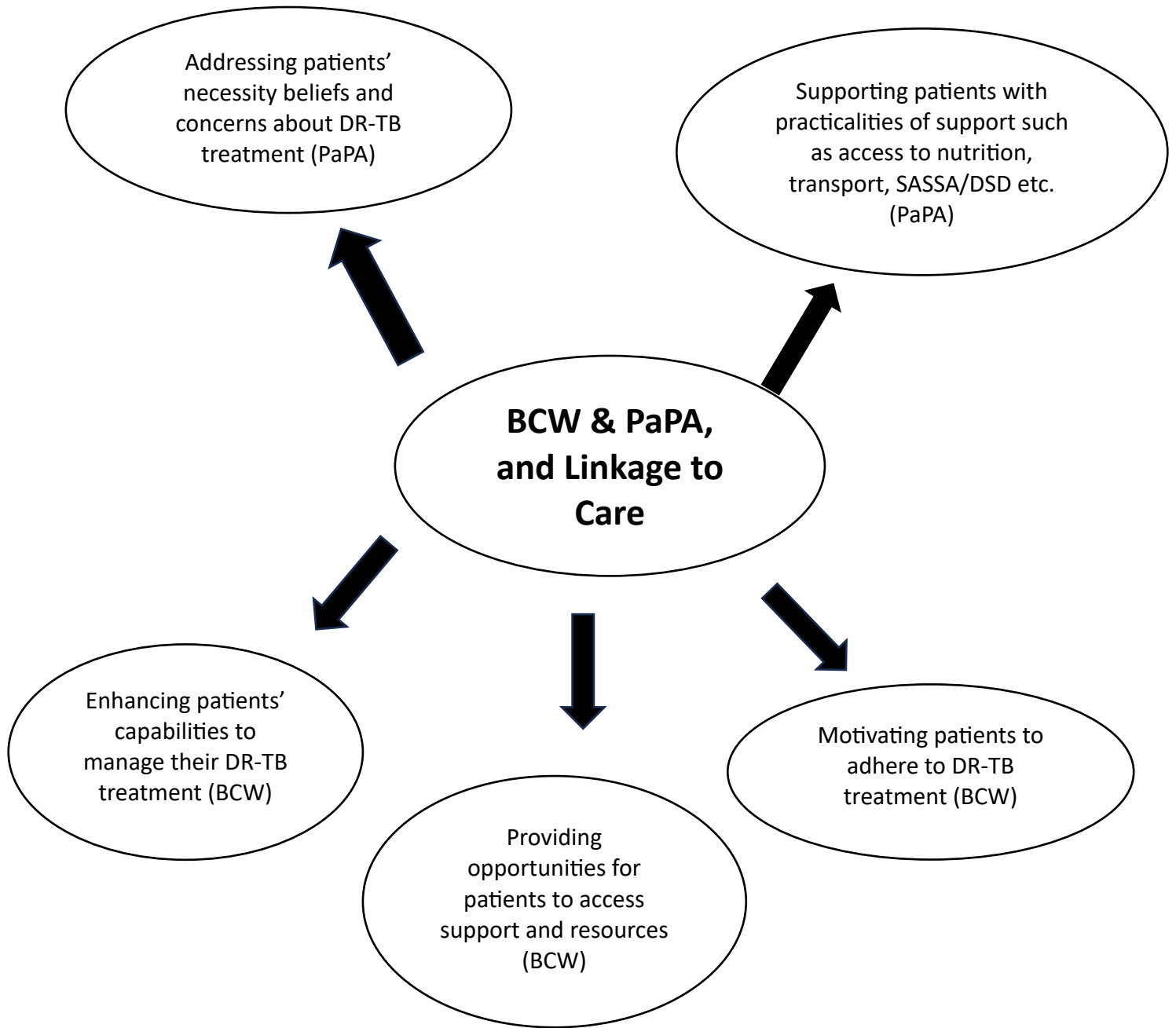

**Figure 3: BCW & PAPA, and Linkage to care**

Supplement: S3 File — (PDF) [file pone.0343154.s006.pdf]

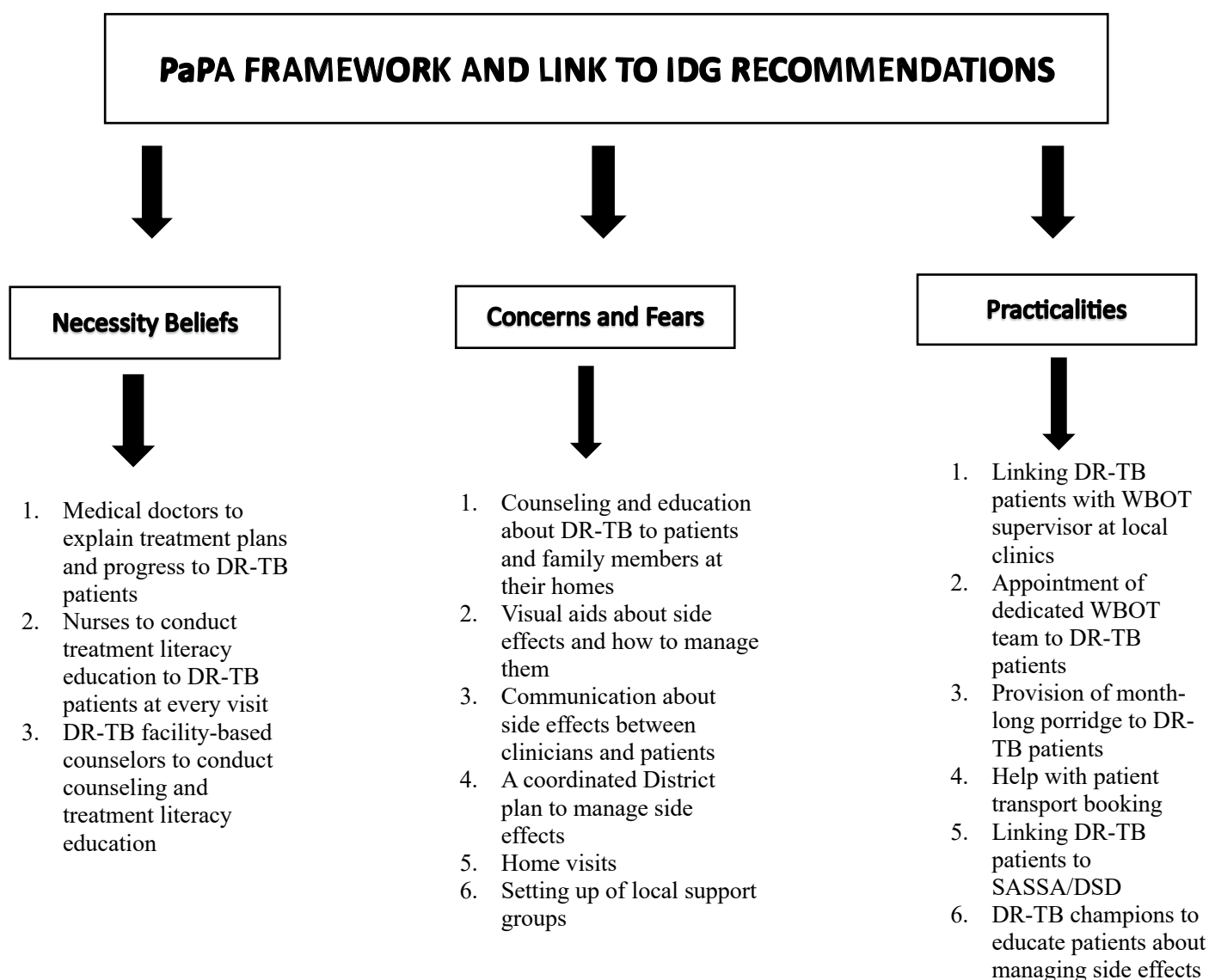

**Figure 5: Figure of PaPA Framework and link to IDG Recommendations**

Supplement: S5 File — (PDF) [file pone.0343154.s008.pdf]
