# Supplementary material for: Designing and evaluating the acceptability of a psychosocial and socioeconomic support package for people with drug-resistant tuberculosis in Johannesburg, South Africa
Source: PLoS One. 2026 Mar 3;21(3):e0343154. doi: 10.1371/journal.pone.0343154 (PMC12956097; doi:10.1371/journal.pone.0343154)
Supplement: S4 File — (PDF) [file pone.0343154.s007.pdf]

BCW FRAMEWORK AND LINK TO IDG RECOMMENDATIONS

Capability

1. Training of WBOTS to conduct counseling sessions
2. DR-TB facilities-based counselors to conduct counseling and treatment literacy education
3. Nurses to conduct treatment literacy education to DR-TB patients
4. Medical doctors to explain treatment plans and progress to DR-TB patients
5. DR-TB centers and local clinics to communicate about patient appointments

Opportunity

1. Linking DR-TB patients with WBOT supervisor at local clinic
2. Appointment of a dedicated WBOT teams to DR-TB patients
3. Setting up local support groups
4. Collaboration between DR-TB facilities and tertiary hospitals on treatment of side effects
5. WBOTS conducting home visits

Motivation

1. Counselling and education about DR-TB to patients and family members at their homes
2. Empowering DR-TB patients and family members to know about DR-TB and its treatment
3. Patient reminders about taking treatment
4. DR-TB champions to share experiences about DR-TB treatment journey
5. Treatment reminders

Figure 4: Figure of BCW Framework and link to IDG recommendations
